# Analysis of Differences in the Classification of Endometrial Cancer Patients in Poland

**DOI:** 10.3390/cancers17020213

**Published:** 2025-01-10

**Authors:** Wiktor Szatkowski, Tomasz Kluz, Małgorzata Cieślak-Steć, Magdalena Śliwińska, Małgorzata Nowak-Jastrząb, Izabela Winkler, Jacek Tomaszewski, Paweł Blecharz

**Affiliations:** 1 Department of Gynaecologic Oncology, Maria Sklodowska-Curie National Research Institute of Oncology Krakow Branch, 31-115 Kraków, Poland; pawel.blecharz@interia.pl; 2 Department of Gynecology and Obstetrics, Institute of Medical Sciences, Medical College, Rzeszow University, 35-025 Rzeszów, Poland; tkluz@ur.edu.pl; 3 Department of III Clinic of Radiotherapy and Chemotherapy, Maria Sklodowska-Curie National Research Institute of Oncology Gliwice Branch, 31-115 Kraków, Poland; malgorzata.cieslak@krakow.nio.gov.pl (M.C.-S.); magdalena.sliwinska@gliwice.nio.gov.pl (M.Ś.); 4 Second Department of Gynecological Oncology, St. John’s Center of Oncology of the Lublin Region, 20-090 Lublin, Poland; ikochans@interia.pl (I.W.); jtomaszewski@cozl.pl (J.T.)

**Keywords:** endometrial cancer, DNA polymerase epsilon, MSI, p53, molecular classification, FIGO 2023

## Abstract

**Background:** Endometrial cancer (EC) incidence and mortality have been steadily rising globally over recent decades. The introduction of advanced molecular technologies, such as next-generation sequencing (NGS) alongside the FIGO 2023 classification, presents opportunities for refined diagnostics and risk stratification. This study aimed to analyze differences in EC classification among oncology centers in southeastern Poland. **Methods:** Data were collected from 461 consecutive patients newly diagnosed with EC between 2022 and 2024 at four major oncology centers in southeastern Poland. Molecular and immunohistochemical (IHC) analyses were conducted on formalin-fixed paraffin-embedded (FFPE) tissues to identify key markers, including POLE mutations, MSI-H, and p53 status. **Results:** The application of the FIGO 2023 staging system revealed statistically significant inter-center differences, with Centers 1 and 4 diagnosing a higher proportion of early-stage cases. The most prevalent subtype was NSMP, observed in 51% of cases. MSI-H occurred in 13–36% of patients, depending on the center. p53 mutations ranged from 9% to 26%. POLE mutations were identified in 4% of patients overall. Significant variations in the molecular subtype distribution across centers highlight potential differences in diagnostic access or tumor biology. **Conclusions:** The findings demonstrate regional differences in EC staging and molecular profiles in Poland, potentially reflecting disparities in diagnostic resources, methodologies, or tumor characteristics. Addressing these variations through standardized diagnostic protocols and equitable access to molecular tools is critical for optimizing patient outcomes. Future research should focus on evaluating the impact of molecular markers on therapy response and prognosis to guide personalized treatment strategies.

## Simple Summary

Endometrial cancer is the most common gynecological malignancy in developed countries with increasing incidence and mortality rates. The introduction of new molecular technologies and the updated 2023 FIGO staging system have revolutionized diagnostics and enabled personalized treatment approaches. In this study, we analyzed clinical and molecular data from patients treated in southeastern Poland to understand the impact of the new FIGO classification on clinical practice. The results demonstrated significant differences in stage assignment and molecular profiles across medical centers, suggesting variations in diagnostic strategies and access to advanced testing methods. These findings emphasize the importance of standardized diagnostic practices and equitable access to molecular testing to ensure consistent implementation of the new FIGO staging system and to improve treatment outcomes for patients with endometrial cancer.

## 1. Introduction

Endometrial cancer (EC) is the most common gynecological malignancy in developed countries, with both its incidence and mortality rates rising in recent years [1]. Between 2015 and 2018, five years after diagnosis, 76% of patients with endometrial cancer in Poland were still alive [2]. This trend highlights the critical need for advancements in diagnostic and therapeutic strategies. The updated 2023 FIGO classification represents a significant milestone by incorporating molecular markers into traditional anatomical staging. These markers, including DNA polymerase epsilon (POLE) mutations, p53 status, and microsatellite instability (MSI-H), are now recognized as key predictive and prognostic factors in EC [3].

Modern EC diagnostics leverage a combination of next-generation sequencing (NGS), Sanger sequencing (PCR), and traditional immunohistochemistry (IHC). The gold standard integrates molecular diagnostics for POLE mutations, mismatch repair (MMR) proteins, and p53 into a comprehensive approach such as the PROMISE algorithm [4]. These technological advancements are pivotal for achieving more precise diagnostic insights and facilitating personalized treatment.

This study represents the first comprehensive analysis of the prevalence of key molecular features of EC in Poland. It evaluates the implementation of the FIGO 2023 classification in oncology centers across southeastern Poland. Specifically, this study aims to carry out the following:

1. Identify differences in staging and molecular subtype classification among centers.
2. Highlight regional disparities in the use of advanced molecular diagnostics.

The findings contribute to the growing body of evidence regarding FIGO 2023’s clinical utility and underscore the importance of diagnostic standardization for optimizing treatment outcomes. By addressing gaps in molecular and clinical practices, this research offers valuable insights for improving patient care both regionally and globally.

## 2. Materials and Methods

### 2.1. Study Design and Population

This study included consecutive patients newly diagnosed with endometrial cancer (EC) between April 2022 and April 2024. The patients were treated at four major gynecologic oncology centers in southeastern Poland, located in the Silesian, Lesser Poland, Subcarpathian, and Lublin Voivodeships. Cases without complete medical records or treated prior to the implementation of the FIGO 2023 classification were excluded. Molecular and immunohistochemical (IHC) analyses were performed on surgical specimens obtained during hysterectomy procedures.

### 2.2. Immunohistochemistry (IHC) Analysis

IHC analyses of MLH1, MSH2, MSH6, PMS2, and p53 proteins were performed uniformly across all centers using OptiView and UltraView kits (Ventana) [Roche Diagnostics, Indianapolis, IN, USA] on the BenchMark Ultra system. Immunohistochemistry was the primary method used for analyzing mismatch repair (MMR) protein status and for p53 evaluation, ensuring consistency in diagnostic approaches.

### 2.3. DNA Extraction

DNA was extracted from formalin-fixed paraffin-embedded (FFPE) tissue samples. Pathologists pre-assessed the tissue blocks to select the most suitable samples for molecular analysis and to evaluate the percentage of tumor cells. The following kits were used for DNA isolation:

- QIAamp DSP DNA FFPE Tissue Kit (Qiagen, Hilden, Germany);
- Maxwell^®^ RSC DNA FFPE Kit and Maxwell^®^ RSC Instrument (Promega, Madison, WI, USA).

### 2.4. Molecular Analysis

POLE Sequencing

Sanger sequencing was conducted on exons 9, 11, 13, and 14 of the POLE gene. In the molecular analyses, POLE gene mutations were evaluated based on the classification proposed by León-Castillo et al. Only mutations identified as pathogenic according to this publication were included [5]. PCR-based techniques were used in cases requiring additional confirmation.

NGS Analysis

Next-generation sequencing (NGS) was performed on the IonTorrent platform using a custom-designed panel (Thermo Fisher Scientific Ion AmpliSeq Designer, https://ampliseq.com/login/login.action, accessed on 8 January 2025). The panel targeted key genes, including MLH1, MSH2, MSH6, PMS2, POLE, and TP53. Sequencing was carried out on CHEF and S5 instruments with Ion 510™, Ion 520™, and Ion 530™ kits (Thermo Fisher Scientific, Waltham, MA, USA).

### 2.5. LVSI Assessment

The criteria for assessing lymphovascular space invasion (LVSI) in this study were based on the WHO definition, which defines substantial LVSI as the involvement of five or more lymphovascular spaces by tumor cells.

### 2.6. Standardization Across Centers

Molecular analyses were conducted uniformly across all participating centers to minimize variability. MMR protein status and p53 were assessed via immunohistochemistry (IHC) at all centers, while POLE mutations were evaluated using molecular techniques (Sanger sequencing and PCR). These methodologies were standardized to ensure consistent application and interpretation across institutions.

### 2.7. Variant Classification

Detected genetic variants were categorized into five classes:

- Pathogenic;
- Likely pathogenic;
- Variants of unknown significance (VUS);
- Likely benign;
- Benign.

Bioinformatics tools and databases, including Cancer Genome Interpreter, Varsome, ClinVar, OncoKB, and Franklin, were utilized for variant classification. Polymorphisms, benign, likely benign, and VUS variants were excluded from the final analysis.

### 2.8. Ethical Considerations

All data were anonymized before analysis to ensure compliance with data protection regulations.

## 3. Results

Clinical, microscopic, and molecular data were collected for 461 patients diagnosed with endometrial cancer (EC) across four oncology centers in southeastern Poland. The mean age of the patients was 65.8 years, with no statistically significant differences observed in mean age between the centers. The histological subtypes of EC varied significantly across centers, as presented in Table 1, with endometrioid carcinoma G1/G2 being the most common subtype overall (77.4%), and there was notable variability in the proportion of aggressive subtypes such as G3 and serous carcinoma.

Molecular analyses revealed that the most common molecular subtype was NSMP, representing 51% of cases (Table 2). Microsatellite instability (MSI-H) was identified in 13–36% of patients depending on the center, while p53 mutations ranged from 9% to 26%. POLE mutations were observed in 4% of all cases, with significant differences between centers (Table 2, *p* = 0.018). These findings indicate the variability in the prevalence of key molecular features, likely reflecting differences in diagnostic approaches.

In terms of pathological features, myometrial invasion showed no statistically significant differences between centers (Table 3, *p* = 0.151). However, substantial LVSI varied significantly, being present in 23–35% of cases depending on the center (Table 4, *p* = 0.008). Similarly, the prevalence of lymph node metastases showed inter-center variability, particularly in para-aortic lymph nodes, where significant differences were observed (Table 4, *p* = 0.016).

Staging according to the FIGO 2023 classification revealed significant inter-center differences (Table 5, *p* = 0.019). Centers 1 and 4 had a higher proportion of early-stage cases (FIGO I), while Centers 2 and 3 reported more advanced-stage cases (FIGO II and III). Substage analysis highlighted that most patients in FIGO I were classified as IA or IB, with relatively few cases classified as IC.

These results collectively highlight the significant inter-center variability in histological subtypes, molecular features, pathological findings, and staging, emphasizing the need for standardized diagnostic protocols to improve comparability and clinical decision-making.

## 4. Discussion

The 2023 FIGO classification for endometrial cancer (EC) marks a significant advancement in personalized medicine by combining traditional anatomical criteria with critical pathological parameters, such as histological subtype, histopathological grade, the presence of lymphovascular space invasion (LVSI), and molecular alterations, including POLE mutations, p53 status, and MSI-H [3]. This integrated approach not only enhances prognostic precision but also reflects the growing emphasis on tailoring treatment strategies to individual patient profiles. It enables the distinction between highly aggressive subtypes—such as serous, clear cell, poorly differentiated endometrioid, mucinous, mesonephric, gastrointestinal type, undifferentiated carcinomas, and carcinosarcomas—and less aggressive subtypes like G1/G2 endometrioid carcinoma.

Furthermore, the inclusion of aggressive features such as LVSI in staging has significantly influenced treatment paradigms, particularly in the adaptation of adjuvant therapies to improve patient outcomes [6,7,8,9,10,11,12]. However, this approach demands robust diagnostic infrastructure and consistent application across healthcare centers to maximize its clinical utility.

Our study revealed substantial inter-center variability in the application of the FIGO 2023 classification, underscoring regional disparities in Poland. Centers 1 and 4 reported a higher proportion of early-stage diagnoses, which may reflect better access to advanced diagnostic tools, such as MRI and molecular testing, as well as greater patient awareness of early symptoms. In contrast, Centers 2 and 3 reported a higher prevalence of advanced cases, likely influenced by limited access to specialized care or delays in diagnosis due to infrastructural constraints. These findings highlight the critical role that healthcare infrastructure, resource allocation, and public health awareness play in determining the clinical stage at diagnosis and subsequent management.

### 4.1. Molecular Profiles

Immunohistochemical (IHC) detection of MMR proteins is still not a standard practice in many centers, particularly those that rely on external pathology laboratories with limited resources [13]. This inconsistency highlights a critical barrier to the uniform application of molecular diagnostics in endometrial cancer.

Our study revealed significant differences in the distribution of molecular subtypes between centers. For instance, p53 mutations were identified in 27% of cases in Center 3 but only in 9% of cases in Center 2. Similarly, the detection rates of molecular markers such as MMR-D varied widely (13–36%), while POLE mutations were uniformly rare at 4%. These discrepancies underscore the absence of standardized diagnostic protocols across the institutions and highlight the significant inter-center variability in molecular diagnostics.

These findings emphasize the importance of standardizing diagnostic protocols to ensure consistency and reliability in molecular profiling. Harmonized approaches would not only enhance diagnostic accuracy but also reduce the risk of misclassification, which can significantly impact treatment decisions and patient outcomes.

### 4.2. Comparison with International Data

Retrospective analyses have consistently demonstrated that patients with POLEmut tumors have the lowest risk of recurrence and mortality among all molecular subtypes [14,15]. However, the rarity of POLEmut cases, the limited number of cases documented in the literature, and the relatively short follow-up periods in many studies pose significant challenges to establishing definitive treatment guidelines [16,17].

When comparing the findings of this study with international benchmarks, notable similarities and discrepancies emerge. The prevalence of POLE mutations in this cohort (4%) is slightly lower than the 7% reported by TCGA [18] but aligns closely with findings from ProMisE studies, which report POLE mutations in 5–12% of cases [4]. Similarly, the NSMP subtype, representing 51% of cases in our cohort, aligns with findings from ProMisE studies, where NSMP accounts for approximately 40–50% of cases [4].

In contrast, significant inter-center variability was observed in the distribution of other molecular subtypes, such as MMR-D and p53abn. For example, MMR-D ranged from 13% to 36%, and p53abn ranged from 9% to 27%, depending on the institution. These differences may reflect regional and methodological variations, including diagnostic practices, resource availability, and the expertise of pathology teams.

This variability underscores the challenges of direct comparisons between studies conducted in different healthcare systems. Misclassification of p53 abnormalities, for instance, can lead to significant clinical consequences, including overtreatment or undertreatment. Overdiagnosis of p53abn may result in assigning patients with superficial invasion to chemoradiotherapy, despite the absence of clear clinical indications, while underestimating its presence may increase the risk of recurrence, particularly in tumors with aggressive molecular profiles [19,20,21,22].

These comparisons highlight the importance of considering regional differences, diagnostic methodologies, and case selection criteria when interpreting molecular data. Additionally, the findings align with Kommoss et al.’s study, which reported MMR-D in 28.1%, POLEmut in 9.3%, p53abn in 12.2%, and NSMP in 50.4% of cases [23]. While our cohort showed a similar prevalence of NSMP (51%), the significant inter-center variability in other subtypes emphasizes the need for standardized diagnostic practices [24,25].

### 4.3. Challenges in Pathological Evaluation

Our findings are consistent with previous studies that have highlighted significant challenges in histopathological evaluation. Retrospective analyses, such as those conducted in the PORTEC-3 trial, revealed reclassification rates as high as 43% following central pathology review, underlining the substantial variability in initial diagnoses [21]. Similarly, Grevenkamp et al. identified critical discrepancies in 9.7% of cases, with direct implications for therapeutic decisions [26]. These discrepancies emphasize how variability in pathological interpretation can result in suboptimal treatment strategies, either through overtreatment or undertreatment.

The observed challenges underscore the necessity for centralized pathology reviews, particularly in complex or borderline cases, to ensure consistency and diagnostic accuracy. Centralized reviews not only reduce variability but also enhance the reliability of staging and molecular profiling, which are critical for tailoring adjuvant therapies.

Additionally, the need for stricter adherence to standardized histopathological and molecular guidelines, such as those outlined by ESGO/ESTRO/ESP, is evident. Such guidelines can help mitigate inter-observer variability and ensure that therapeutic decisions are based on accurate and reproducible diagnostic data. Incorporating regular training programs and quality assurance initiatives for pathologists could further enhance diagnostic consistency across institutions.

### 4.4. Standardization Issues in LVSI Assessment

Defining significant LVSI involvement remains inconsistent across international guidelines [27]. FIGO 2023, WHO 2020, and ESGO/ESTRO/ESP guidelines define substantial LVSI as five or more affected lymphovascular spaces, while the NCCN uses a slightly different threshold, requiring four spaces on a single H&E slide to indicate substantial LVSI [27]. These discrepancies highlight the lack of a universally accepted definition, creating challenges in both clinical practice and research. The criteria for assessing lymphovascular space invasion (LVSI) in this study were based on the WHO definition, which defines substantial LVSI as the involvement of five or more lymphovascular spaces by tumor cells.

The intraclass correlation coefficient (ICC) for LVSI assessment, as reported by Turashvili et al., is only 0.6 for the presence of LVSI and 0.56 for “substantial” LVSI [28]. This moderate level of agreement underscores the inherent subjectivity in LVSI evaluation, even among experienced pathologists [29,30]. Variability in interpretation not only complicates accurate staging but also introduces the risk of “stage drift,” where differences in how LVSI is assessed may lead to inconsistencies in assigning patients to specific treatment protocols.

Such variability in LVSI assessment limits the comparability of treatment outcomes and prognostic evaluations between institutions. For example, patients classified as having substantial LVSI under one guideline might not meet the criteria under another, potentially leading to differences in adjuvant treatment recommendations. Standardization of LVSI assessment criteria, coupled with enhanced training and quality control measures for pathologists, is essential to minimize variability and ensure consistent clinical decision-making.

### 4.5. Future Directions

This study underscores the urgent need for the standardization of diagnostic protocols and equitable access to advanced molecular diagnostic tools across all centers. The current variability in molecular testing and pathological evaluation not only hinders consistent classification but also limits the ability to implement personalized treatment strategies effectively. Establishing centralized pathology reviews and adopting uniform diagnostic criteria, such as the PROMISE algorithm, would significantly enhance the consistency of endometrial cancer (EC) classification and management.

Future research should prioritize investigating the prognostic and predictive value of key molecular markers, including p53, MSI-H, and POLE mutations. Understanding how these markers influence treatment responses and clinical outcomes could provide a foundation for developing more precise therapeutic strategies tailored to specific molecular subtypes. For instance, further exploration of the mechanisms underlying the low recurrence rates and mortality in POLEmut tumors or the aggressive nature of p53abn tumors could inform treatment de-escalation or intensification protocols.

Additionally, international collaborations could play a pivotal role in refining and validating molecular classification systems. Harmonizing global diagnostic standards would not only reduce inter-center variability but also improve the comparability of clinical trial results and facilitate the integration of novel biomarkers into routine clinical practice. Expanding research efforts to include diverse populations and healthcare systems will ensure that future advances are broadly applicable, ultimately improving outcomes for EC patients worldwide.

### 4.6. Strengths and Limitations

This study is the first to examine molecular profiles of endometrial cancer (EC) specifically within the Polish population, filling a significant gap in the existing literature. The geographically coherent dataset and the inclusion of consecutive, unselected cases from multiple centers enhance the study’s reliability and provide a comprehensive overview of EC in this region. By reflecting real-world clinical settings, this study offers valuable insights into the practical challenges and variability associated with molecular diagnostics.

However, several limitations should be noted. Variability in genetic testing methods and the combination of molecular and immunohistochemical approaches across participating centers may have introduced inconsistencies in molecular subtype classification. This heterogeneity, while representative of actual clinical practice, highlights the need for greater methodological standardization to ensure more uniform and comparable results in future research.

Additionally, the absence of a centralized pathology review may have affected the consistency of histopathological and molecular evaluations, potentially contributing to inter-center variability. The relatively small cohort size for certain molecular subtypes, such as POLEmut, further limits the generalizability of the findings and underscores the need for larger, multicenter studies to validate these results.

It is important to note that the primary aim of this study was not to resolve the existing disparities in diagnostic practices across oncology centers but rather to highlight their presence and potential impact on patient care. By demonstrating the variability in diagnostic methodologies and the resulting differences in molecular and pathological classifications, we aim to provide a foundation for further research and discussions. This study underscores the need for standardized diagnostic protocols and equitable access to molecular testing, but it does not seek to propose definitive solutions to these challenges. Instead, our findings serve as a call to action for collaborative efforts to address these issues in future studies.

Despite these limitations, the study provides a critical foundation for understanding the molecular landscape of EC in Poland and emphasizes the importance of standardizing diagnostic practices to improve the accuracy and utility of molecular classification.

## 5. Conclusions

This study highlights significant variability in the application of the FIGO 2023 classification for endometrial cancer (EC), particularly in staging and molecular profiling, across oncology centers in southeastern Poland. These findings reveal critical disparities in diagnostic practices, reflecting differences in resource availability, diagnostic methodologies, and adherence to guidelines.

The observed variability underscores the urgent need for the standardization of diagnostic protocols and equitable access to advanced molecular diagnostic tools. Uniformity in diagnostic approaches, including centralized pathology reviews and the adoption of standardized molecular frameworks such as the PROMISE algorithm, is essential to enhance the reliability and comparability of EC classification across institutions. Such measures would ensure the full clinical utility of the FIGO 2023 classification and facilitate more accurate prognostication and treatment stratification.

Future research should focus on evaluating the prognostic and predictive value of molecular markers, including POLE, p53, and MSI-H, in diverse patient populations. These investigations will provide critical insights into the relationship between molecular profiles and treatment responses, guiding the development of more personalized therapeutic strategies. By addressing these gaps, ongoing efforts can improve outcomes for EC patients both in Poland and globally, paving the way for more consistent and equitable cancer care.

## Figures and Tables

**Table 1:** Analysis of histological types in individual centers.

	Center 1		Center2		Center 3		Center 4		Total		Statistical Significance *p*
	N	%	N	%	N	%	N	%	N	%	
**Non-aggressive types**											*p* < 0.001
Endometrioid carcinoma G1/G2	69	74.2	79	62.7	53	76.8	156	90.2	357	77.4	
**Aggressive types**											
Endometrioid carcinoma G3	11	11.8	35	27.9	9	13.0	7	4.0	62	13.4	
Serous carcinoma	7	7.6	7	5.5	4	5.9	3	1.8	21	4.5	
Other	6	6.4	5	3.9	3	4.3	7	4.0	22	4.7	

**Table 2:** Analysis of molecular features by individual centers.

	Center 1		Center 2		Center 3		Center 4		Total		*p*
**NGS/Sanger/IHC**	**N**	**%**	**N**	**%**	**N**	**%**	**N**	**%**	**N**	**%**	
**POLE**	7	7	4	3	3	4	7	4	21	4	*p* = 0.018
**MMRd**	23	24	45	36	9	13	49	28	126	27	
**p53**	18	19	11	9	17	26	31	18	77	18	
**NSMP**	45	50	66	52	40	57	86	50	227	51	
	93	100	126	100	69	100	173	100	461	100	

**Table 3:** Analysis of myometrial and cervical stroma invasion by individual centers.

	Center 1		Center 2		Center 3		Center 4		Total		Statistical Significance *p*
	N	%	N	%	N	%	N	%	N	%	
**Myometrial invasion**											*p* = 0.151
Limited to the endometrium	4	4.3	3	2.3	6	8.7	6	3.5	19	4.1	
Invasion of less than half	46	49.5	60	47.6	33	47.8	92	53.2	231	50.0	
Invasion of half or more	40	43.0	63	50.0	27	39.1	67	38.7	197	42.7	
Invasion of uterine serosa	3	3.2	0	0	3	4.3	8	4.6	14	3.0	
Invasion of cervical stroma	14	15.1	24	19.1	7	10.1	33	19.1	68	14.7	*p* = 0.326

**Table 4:** Analysis of LVSI and lymph node involvement by individual centers.

	Center 1		Center 2		Center 3		Center 4		Total		Statistical Significance *p*
	N	%	N	%	N	%	N	%	N	%	
**LVSI**											*p* = 0.008
Substantial LVSI	28	30.1	34	26.9	16	23.2	61	35.3	139	30.1	
Focal LVSI	10	10.7	13	10.3	4	5.8	2	1.2	29	6.3	
**Metastasis to the pelvic lymph nodes**											*p* = 0.413
Macrometastasis	8	8.6	7	5.6	4	5.8	14	8.1	33	7.2	
Micrometastasis	2	2.2	1	0.8	0	0	0	0	2	0.4	
**Metastasis to para-aortic lymph nodes**											*p* = 0.016
Macrometastasis	5	5.4	2	1.6	1	1.5	0	0	8	1.7	
Micrometastasis	0	0	0	0	0	0	0	0	0	0	

**Table 5:** Distribution of FIGO 2023 staging across individual centers.

	Center 1		Center 2		Center 3		Center 4		Total		Statistical Significance *p*
	N	%	N	%	N	%	N	%	N	%	
**I**	57	61	68	55	33	48	81	47	239	52	*p* = 0.019
**IA**	40	44	46	35	19	27	68	40			
**IB**	17	18	21	17	12	17	12	6			
**IC**	0	0	1	1	2	3	1	0.5			
**II**	13	14	39	30	28	40	62	36	142	31	
**IIA**	4	4	4	3	3	4	13	7			
**IIB**	1	1	12	10	7	10	26	15			
**IIC**	8	9	23	20	18	26	23	13			
**III**	21	22	17	13	7	10	28	16	73	16	
**IIIA**	5	6	3	2	2	3	4	2			
**IIIB**	1	1	4	3	2	3	9	5			
**IIIC**	15	16	10	8	3	4	15	8			
**IV**	2	3	2	2	1	2	2	1	7	1	
**IVA**	2	2	0	0	0	0	0	0			
**IVB**	0	0	2	2	1	1	2	1			
Total	93	100	126	100	69	100	173	100	461	100	

## Data Availability

The original contributions presented in this study are included in the article. Further inquiries can be directed to the corresponding author(s).
